# New developments and prospects for drug delivery in medulloblastoma

**DOI:** 10.1016/j.omton.2026.201186

**Published:** 2026-03-25

**Authors:** Kaoutar Bentayebi, Sara Louati, Keittisak Suwan, Rachid Eljaoudi, Amin Hajitou

**Affiliations:** 1 Cancer Phage Therapy Laboratory, Department of Brain Sciences, Imperial College London, London, UK; 2 Medical Biotechnology Laboratory, Faculty of Medicine and Pharmacy, University Mohammed V, Rabat, Morocco

**Keywords:** MT: Special Issue - Advancements in pediatric cancer therapy, compartment-matched delivery, preclinical models, translational science, drug transport, gene therapy, central nervous system

## Abstract

Medulloblastoma accounts for approximately 20% of all pediatric brain tumors. Standard treatment typically involves a multimodal approach including surgery, radiation therapy, and chemotherapy. Despite advances in molecular classification and the development of targeted therapies, long-term survival and quality of life remain suboptimal, particularly for patients with high-risk subgroups. Although targeted therapies hold significant promise, their effective delivery to the brain remains a major challenge. The unique cerebellar microenvironment, including the blood-brain barrier, the blood-brain-tumor barrier, and the blood-cerebrospinal fluid barrier, restricts drug penetration and therapeutic efficacy. To overcome these limitations, innovative strategies are being explored to bypass or transiently modulate these barriers in a subgroup- and compartment-specific manner. These approaches include nanoparticle-based delivery systems, focused ultrasound, intrathecal administration, and rational combination therapies. Emerging platforms such as transmorphic phage/adeno-associated virus vectors and lysosomal targeting strategies are under active investigation and may further enhance therapeutic precision. This review provides a comprehensive overview of current brain-targeted drug delivery systems for medulloblastoma, highlighting barrier heterogeneity, recurrence patterns, and emerging technologies with the potential to improve therapeutic efficacy and long-term outcomes for children affected by this aggressive pediatric brain tumor.

## Graphical abstract

**Figure:**
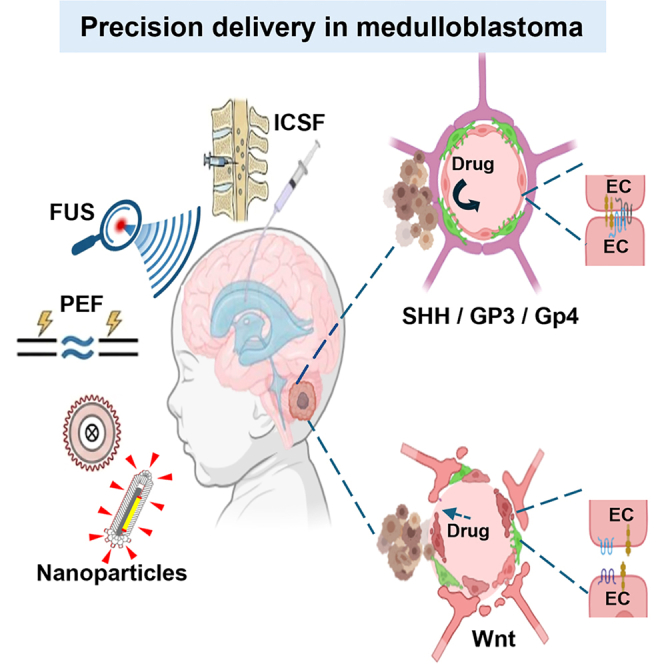

## Teaser

Medulloblastoma subgroups exhibit distinct blood-brain and blood-tumor barrier characteristics that influence therapeutic exposure and patterns of failure. This review examines biologic barriers and emerging delivery technologies, emphasizing the need to consider barrier heterogeneity when developing strategies for pediatric brain tumor treatment.

## Introduction

Brain tumors are among the most severe and lethal cancers affecting both pediatric and adult populations. In children, brain tumors are the leading cause of cancer-related mortality, accounting for approximately 40% of all pediatric cancer deaths. These tumors are associated with high morbidity, significantly affecting quality of life through neurological deficits and cognitive impairment.^1^^,^^2^^,^^3^ Medulloblastoma (MB) is the most common malignant brain tumor in children, representing approximately 20% of all childhood brain tumors.^4^ Originating in the cerebellum, MB frequently presents with balance disturbances, incoordination, and symptoms of hydrocephalus. Its propensity to disseminate through the cerebrospinal fluid (CSF) to other central nervous system (CNS) sites significantly impacts prognosis.^5^

While multimodal protocols incorporating surgery, radiotherapy, and chemotherapy have significantly improved survival, the heavy burden of craniospinal irradiation and intensive systemic regimens continues to inflict profound long-term neurological and neuroendocrine sequelae.^6^ To address interpatient heterogeneity in outcome, MB has been stratified into four principal molecular subgroups: WNT-activated, Sonic Hedgehog (SHH), group 3, and group 4. This molecular framework has transformed risk stratification and informed subgroup-adapted therapeutic strategies.^7^

Importantly, molecular subgroup identity is largely preserved at recurrence and is associated with reproducible, subgroup-specific anatomical patterns of failure.^8^ Longitudinal clinical and molecular profiling studies demonstrate that SHH MBs predominantly recur locally within the posterior fossa or tumor bed, whereas group 3 and group 4 tumors more frequently relapse with metastatic or leptomeningeal dissemination rather than isolated local recurrence.^8^ These patterns suggest that therapeutic failure is often driven by inadequate drug exposure within the relevant anatomical compartment brain parenchyma for locally recurring disease versus CSF and leptomeningeal spaces for disseminated disease, rather than by intrinsic resistance alone.

Although immunotherapeutic strategies such as immune checkpoint inhibitors and chimeric antigen receptor T cell therapies have shown promise in other malignancies, their efficacy in MB remains limited. This reflects not only an immunosuppressive tumor microenvironment but also restricted therapeutic access imposed by CNS barriers, including the blood-brain barrier (BBB), blood-brain-tumor barrier (BBTB), and blood-CSF barrier (BCSFB).^9^^,^^10^

Barrier integrity in MB is highly subgroup dependent. Preclinical and translational studies demonstrate that WNT-activated tumors exhibit a relatively permeable vasculature, whereas SHH and group 4 tumors frequently retain restrictive BBB features, with group 3 tumors showing intermediate disruption. Notably, even in subgroups with partial BBTB permeability, local recurrence remains common, indicating that heterogeneous or incomplete barrier disruption is insufficient to ensure durable therapeutic exposure.^11^

## Biologic barriers to effective MB treatment

**Figure 1:**
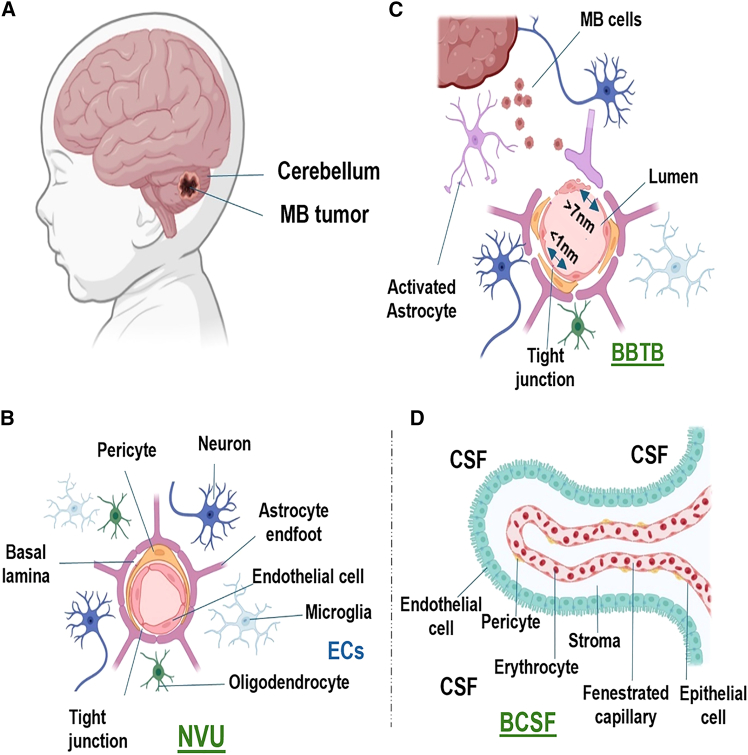
Schematic of barriers limiting drug delivery in MB (A) Anatomical localization of MB within the cerebellum. (B) Cellular and structural components of the neurovascular unit (NVU) and extracellular space (ECS). (C) Structural features of the disrupted blood-brain-tumor barrier (BBTB), depicting impaired tight junctions, astrocyte endfoot detachment, endothelial cell loss, pericyte distribution, and basement membrane degradation. (D) Components of the blood-cerebrospinal fluid barrier (BCSFB) regulating molecular entry into the ventricular system.

Successful pharmacologic treatment of MB is fundamentally constrained by specialized CNS barriers that regulate drug distribution to distinct anatomical compartments. As shown in Figure 1, three physiologically and functionally distinct barriers are particularly relevant: the BBB, the BBTB, and the BCSFB. Failure to distinguish between these barriers during therapeutic development has contributed to limited translational success of many systemic therapies targeting CNS malignancies.^12^^,^^13^^,^^14^

**Figure 2:**
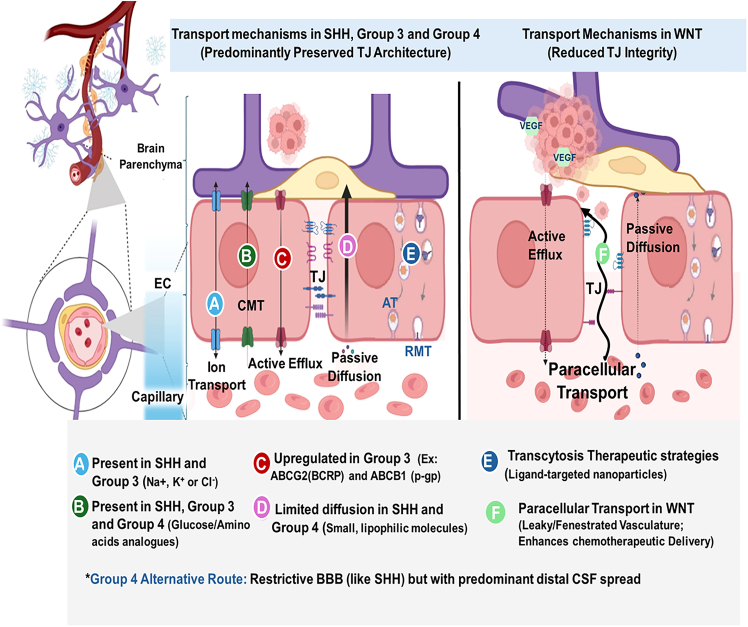
Molecular subgroup-specific barrier heterogeneity in MB Schematic comparison of the NVU across MB molecular subgroups, highlighting differential transport mechanisms. (Left) Depiction of transport in SHH, group 3, and group 4 tumors with predominantly preserved tight junction (TJ) architecture. (Right) Depiction of transport in WNT tumors with reduced TJ integrity. The following features are annotated: A, ion transport; B, carrier-mediated transport (CMT); C, active efflux; D, passive diffusion; E, receptor-mediated transcytosis (RMT) and adsorptive transcytosis (AT); F, paracellular transport in WNT tumors. VEGF, vascular endothelial growth factor.

The BBB is formed by specialized endothelial cells interconnected by tight junctions and supported by pericytes and astrocytic endfeet, creating a highly selective interface with minimal paracellular permeability and low rates of transcytosis (Figure 2). High expression of ATP-binding cassette (ABC) efflux transporters, including P-glycoprotein (ABCB1) and breast cancer resistance protein (BCRP/ABCG2), further limits drug accumulation within the brain. As a result, more than 98% of small-molecule therapeutics fail to achieve therapeutic concentrations within normal brain parenchyma following systemic administration.^15^

Importantly, even in subgroups where partial BBTB permeability is observed, therapeutic failure remains common. SHH MBs frequently recur locally within the posterior fossa despite evidence of heterogeneous vascular leakiness, indicating that incomplete BBTB disruption, limited intratumoral distribution, and insufficient duration of drug exposure can constrain therapeutic efficacy. These observations show that BBB or BBTB leakiness alone is insufficient to ensure tumor eradication.^11^

In contrast to the BBB and BBTB, the BCSFB, primarily located at the choroid plexus epithelium, regulates molecular exchange between the systemic circulation and the CSF. The BCSFB differs substantially from the BBB in cellular composition, transporter expression, permeability, and pharmacokinetic behavior. Consequently, drug concentrations achieved within the CSF cannot be reliably inferred from plasma levels or brain parenchymal exposure.^16^^,^^17^^,^^18^

This distinction is particularly relevant for group 3 and group 4 MBs, which are significantly more likely to relapse with metastatic or leptomeningeal dissemination rather than isolated local failure.^8^ These recurrence patterns strongly suggest that inadequate therapeutic exposure within the CSF and subarachnoid compartments, rather than insufficient parenchymal penetration alone, is a dominant mechanism of treatment failure in these subgroups. Delivery strategies focused exclusively on BBB penetration may therefore fail to address the primary site of residual disease.

Taken together, MB exemplifies a disease in which barrier biology, molecular subgroup identity, and recurrence anatomy are tightly interconnected. Effective drug delivery, therefore, requires compartment-matched strategies that explicitly distinguish between parenchymal and CSF targets rather than assuming uniform CNS exposure. This framework provides a biologically grounded rationale for tailoring delivery approaches to subgroup-specific patterns of failure and for selecting anatomically relevant pharmacokinetic endpoints in preclinical and clinical studies.

## Molecular subgroups of MB and implications for drug delivery

MB is composed of four principal molecular subgroups, each defined by distinct genetic drivers, clinical behavior, and therapeutic vulnerabilities. Beyond their prognostic value, molecular subgroup identities have direct implications for drug delivery, as they influence vascular architecture, BBB integrity, patterns of recurrence, and pharmacologic exposure within the CNS.^11^^,^^19^

### WNT-activated MB

WNT-activated MB accounts for approximately 10% of cases and is associated with excellent clinical outcomes.^19^ A defining delivery-relevant feature of this subgroup is its uniquely permeable tumor vasculature, which distinguishes it from the restrictive barriers found in groups 3 and 4. This leaky phenotype is driven by the tumor’s secretion of Wnt antagonists like WIF1, which suppress canonical BBB signaling in local endothelial cells.^11^

Preclinical studies demonstrate that these vessels exhibit an aberrant, non-CNS-like profile (SLC2A1−/Glut1− and PLVAP+), resulting in significant vascular fenestrations (60- to 80-nm pores) and disrupted tight junctions.^11^

Clinically, this manifests as solid, homogeneous contrast enhancement on MRI, indicating that systemically administered therapeutics can achieve high intraparenchymal concentrations without specialized delivery vehicles. This permissive vascular phenotype likely accounts for the favorable therapeutic responses observed even with reduced-intensity regimens.^11^^,^^20^^,^^21^^,^^22^

However, a critical delivery insight emerges from the rare instances of treatment failure: WNT recurrences frequently involve the metastatic compartment rather than the primary tumor bed.^8^ This suggests that while the leaky primary tumor is easily eradicated by systemic drugs, the CSF may still act as a sanctuary site where drug concentrations remain sub-therapeutic. From a drug-delivery perspective, WNT MB serves as a proof of concept for the efficacy of high parenchymal exposure, but it also highlights that leptomeningeal coverage remains a secondary, yet vital, priority even in the most permeable subgroups.^8^^,^^11^^,^^20^

### SHH MB

SHH-activated MB accounts for approximately 30% of cases and occurs across pediatric and adult populations.^23^ In contrast to WNT tumors, SHH MBs frequently retain restrictive BBB or BBTB characteristics that limit passive diffusion of systemic agents.^11^ Clinically, SHH tumors demonstrate a strong propensity for local recurrence within the posterior fossa or tumor bed rather than widespread metastatic dissemination.^8^

The restricted delivery observed in SHH MB is reinforced by a subgroup-specific immuno-stromal niche. Unlike the leaky WNT subgroup, SHH tumors are characterized by a high density of fibroblasts and tumor-associated macrophages, alongside dense extracellular matrix components such as collagens and laminin.^24^ Collectively, these features create an interstitial transport barrier that limits the homogeneous distribution and retention of systemically administered drugs within the tumor tissue.^11^ Consequently, SHH MB represents a prime candidate for delivery strategies specifically designed to enhance local parenchymal exposure. These include physical BBB modulation approaches, biologically targeted nanoparticle systems, and tumor-directed gene delivery platforms capable of bypassing or exploiting microenvironment-specific features that limit conventional drug penetration.^3^^,^^25^^,^^26^

### Group 3 MB

Group 3 MB is associated with aggressive clinical behavior, high rates of metastatic disease at diagnosis, and poor overall prognosis.^27^ Relapse in group 3 disease most frequently occurs in metastatic or leptomeningeal compartments, implicating inadequate therapeutic coverage of the CSF rather than failure of local tumor control alone.^8^

At the molecular level, group 3 tumors exhibit additional features that restrict effective drug exposure, including overexpression of ABC efflux transporters.^28^ Transcriptomic and immunohistochemical analyses demonstrate increased expression of ABCG2 (BCRP) and multiple ABCC family members, including MRP1 (ABCC1), MRP4 (ABCC4), MRP5 (ABCC5), and MRP7 (ABCC10), in group 3 MB. These transporters actively reduce intracellular drug accumulation and contribute to significant pharmacoresistance.^28^^,^^29^

These biological features are especially critical when considering the clinical behavior of this subgroup. Meta-analysis of large patient cohorts confirms that group 3 (30%) and group 4 (31%) tumors have the highest frequency of metastatic disease at diagnosis, with rates rising to 47% in infants.^29^ Taken together, these findings strongly support prioritizing delivery strategies that achieve sustained exposure within the CSF, while circumventing transporter-mediated efflux. Such approaches include intrathecal or intraventricular delivery, CSF-retentive nanocarriers, and biologically targeted gene-delivery platforms capable of bypassing conventional pharmacokinetic limitations.^8^^,^^30^

### Group 4 MB

Group 4 MB is the most prevalent subgroup, accounting for approximately 35%–40% of cases.^31^ Although historically less well defined at the molecular level, group 4 tumors exhibit distinct clinical and biological behavior.^32^ Importantly, group 4 MB often retain intact BBB features compared with other subgroups.^33^

Unlike WNT tumors, group 4 vasculature lacks significant fenestration, maintaining a restrictive barrier that limits passive diffusion of systemic agents.^11^^,^^33^ Clinically, group 4 disease is characterized by a high incidence of metastatic and leptomeningeal relapse. This combination of restrictive parenchymal barrier properties and CSF-dominant patterns of failure presents a dual delivery challenge that cannot be addressed by strategies focused solely on BBB penetration.^29^

Collectively, these subgroup-specific differences highlight the limitations of one-size-fits-all delivery approaches in MB. Effective therapeutic strategies must integrate molecular subgroup biology, dominant anatomical sites of failure, and barrier-specific constraints to achieve clinically meaningful and durable drug exposure.

### Preclinical models of MB and their implications for drug delivery

**Figure 3:**
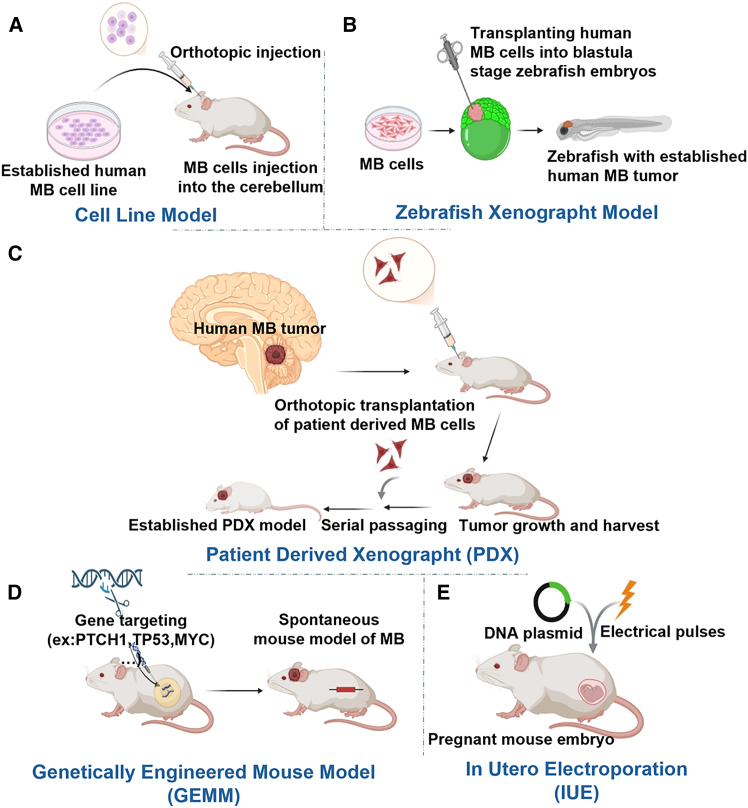
Preclinical modeling strategies for medulloblastoma (A) *In vitro* cell line models. (B) Zebrafish xenograft models involving embryo transplantation. (C) Patient-derived xenograft (PDX) models in immunodeficient mice. (D) Genetically engineered mouse models (GEMMs) harboring specific germline or somatic mutations. (E) *In utero* electroporation (IUE) models depicting genetic modification of the developing cerebellum.

Preclinical model systems are key for evaluating emerging therapeutic strategies in MB as shown in Table S1^34^^,^^35^^,^^36^^,^^37^^,^^38^^,^^39^^,^^40^^,^^41^^,^^42^^,^^43^^,^^44^^,^^45^^,^^46^^,^^47^^,^^48^^,^^49^^,^^50^ and Figure 3. However, their translational relevance critically depends on how accurately they recapitulate clinically relevant drug delivery barriers, anatomical compartments, and patterns of disease spread.^51^^,^^52^ Studies focusing exclusively on tumor reduction or survival often overlook compartmental pharmacokinetics and target-site pharmacodynamics. These fundamental limitations are what ultimately drive clinical failure in CNS malignancies.^53^^,^^54^^,^^55^

#### Genetically engineered mouse models

Genetically engineered mouse models (GEMMs) provide a critical translational framework by faithfully recapitulating human disease in both genotype and phenotype through native physiological and biochemical pathways.^56^ Unlike xenograft systems, which often utilize immune-compromised hosts, GEMMs preserve the interactions between tumor cells and the microenvironment, including the host vasculature.^57^ This preservation ensures the integrity of the neurovascular unit (NVU) enabling an accurate assessment of intraparenchymal drug penetration under biologically relevant conditions.^11^^,^^21^

These features render SHH GEMMs particularly well suited for interrogating intraparenchymal drug delivery, spatial heterogeneity of therapeutic distribution within posterior fossa tumors, and the durability of local drug exposure. This is especially relevant given that SHH MBs predominantly recur locally, indicating that therapeutic failure is frequently driven by insufficient parenchymal drug penetration or retention rather than inadequate CSF exposure.^8^

#### Patient-derived xenograft models

Patient-derived xenograft (PDX) models preserve human tumor heterogeneity and molecular subgroup identity, making them valuable tools for evaluating therapeutic response across MB subgroups.^58^ However, host genetic background and immunodeficiency strain may influence the interpretation of delivery and efficacy by affecting systemic pharmacokinetics and BBB-related transporter programs, including ABC transporters such as P-glycoprotein.^59^^,^^60^^,^^61^ Furthermore, orthotopic transplantation of PDX tumors has been shown to compromise BBTB integrity compared with autochthonous genetically engineered models, potentially overestimating drug penetration and yielding false-positive efficacy results.^21^

When paired with rigorous compartment-specific pharmacokinetic analyses, PDX-based platforms can provide valuable insight into subgroup-specific therapeutic vulnerabilities in MB, as demonstrated by patient and PDX-derived tumoroid models that preserve molecular subgroup identity and reveal subtype-specific drug sensitivities.^62^ However, Because orthotopic transplantation models, including PDX, can exhibit altered BBB/BBTB integrity, apparent therapeutic efficacy may, in part, reflect enhanced drug access rather than intrinsic tumor sensitivity. Accordingly, interpretation of efficacy data from PDX studies requires consideration of barrier status and is strengthened by pharmacologic measurements of drug exposure within tumor and brain tissue.^21^^,^^63^

#### Models for group 3 and group 4 MBs

Group 3 and group 4 MBs are characterized by a high incidence of distant and leptomeningeal relapse, with metastatic dissemination along the neuraxis representing the dominant pattern of treatment failure.^64^ In these subgroups, relapse frequently involves CSF and spinal compartments rather than isolated recurrence at the primary tumor bed.^64^ Consequently, conventional whole-brain homogenate drug measurements or posterior fossa-restricted pharmacokinetic analyses may inadequately represent drug exposure at clinically relevant metastatic sites.

Given the predominance of leptomeningeal disease at relapse, preclinical evaluation in group 3 and group 4 MBs should incorporate pharmacokinetic and pharmacodynamic assessments within the CSF, spinal leptomeninges, and disseminated tumor niches.^65^^,^^66^ Relevant endpoints include drug concentration, residence time, spatial distribution, and biological activity within metastatic compartments, rather than reliance solely on plasma levels or parenchymal exposure as surrogate measures of efficacy in disseminated disease.^66^

Orthotopic and dissemination competent preclinical models, including metastatic group 3 xenografts and neuraxis-spreading transplantation systems, recapitulate key features of leptomeningeal dissemination observed in patients.^67^ These models are essential for evaluating intrathecal, intraventricular, and CSF-directed delivery strategies under conditions that reflect clinically dominant patterns of failure.^65^^,^^67^

Proper preclinical testing in MB, therefore, requires aligning model selection and pharmacologic endpoints with the anatomical compartment most relevant to recurrence. Integrating molecular subgroup identity, barrier biology, and relapse anatomy into experimental design is critical to improving the translational predictive value of drug delivery studies in MB.

#### Advanced drug delivery technologies for MB

Navigating the unique anatomical and biological barriers of MB requires a repertoire of advanced drug delivery technologies tailored to the subgroup-specific site of disease. Rather than relying on non-specific systemic administration, these modalities enable precise targeting of both the primary tumor mass and the leptomeningeal space. As summarized in Table S2, recent clinical trials have begun to evaluate these prominent approaches, reflecting a shift toward more sophisticated delivery strategies. Physical interventions, such as focused ultrasound (FUS) and pulsed electric fields (PEFs), provide localized parenchymal access by transiently modulating the NVU, whereas direct intracerebrospinal administration bypasses the BBB entirely to treat the neuraxis. Complementing these physical approaches, engineered nanoparticles, including ligand-directed liposomes and RGD4C-targeted vectors (transmorphic phage/AAV [TPA]) utilize molecular recognition to facilitate transport across the NVU and ensure preferential uptake by malignant cells. This high-resolution targeting allows for the delivery of potent therapeutics directly to the tumor microenvironment. By selecting a delivery protocol that accounts for the inherent interpatient heterogeneity, therapeutic concentrations can be achieved at the target site while minimizing the debilitating off-target toxicities of conventional regimens.

#### Intrathecal and intraventricular drug delivery

Intrathecal and intraventricular drug deliveries provide direct access to the CSF, thereby bypassing systemic entry constraints imposed by the BBB and BCSFB. This approach enables the achievement of high local drug concentrations within the leptomeningeal compartment, while limiting systemic exposure and associated toxicity.^68^^,^^69^ In MB, these routes are particularly relevant for group 3 and group 4 diseases, which demonstrate a strong propensity for metastatic and leptomeningeal dissemination along the neuraxis.^8^^,^^11^

Historically, intrathecal chemotherapy has been used primarily in relapsed or refractory leptomeningeal MB, most commonly administered via an Ommaya reservoir, a surgically implanted ventricular catheter system that allows repeated, controlled access to the ventricular CSF space without repeated lumbar puncture. Early-phase clinical studies have evaluated intraventricular administration of agents such as methotrexate and etoposide in this context.^70^^,^^71^^,^^72^ Although technically feasible, durable clinical benefit has been inconsistent, and no standardized intrathecal regimen has been established for MB.

One important limitation of CSF directed therapy is the relatively rapid turnover of human CSF, which reduces drug residence time and contributes to declining intrathecal drug concentrations over time.^69^ Furthermore, CSF-administered agents generally penetrate only a few millimeters into adjacent brain parenchyma, limiting effectiveness against bulky or nodular metastatic deposits.^68^

Emerging insights into CSF interstitial exchange pathways further contextualize these limitations. The glymphatic system, which utilizes perivascular spaces (PVSs) to facilitate CSF interstitial fluid (ISF) exchange, has been proposed as a route for macromolecular transport within the CNS.^73^ Longitudinal imaging studies in MB patients demonstrate that radiotherapy dynamically alters PVS volume, likely reflecting treatment-induced BBB disruption and changes in CSF-ISF exchange.^74^ These findings indicate that barrier and perivascular physiology in MB is not static but evolves during therapy, potentially influencing the distribution and retention of CSF-delivered agents.

Despite historical constraints, the predominance of leptomeningeal relapse in group 3 and group 4 MBs, provides a strong biological rationale for revisiting CSF-directed strategies using modern delivery technologies. Sustained-release formulations, CSF-retentive nanoparticles, and advanced infusion systems aim to prolong residence time and improve spatial coverage across the neuraxis.^68^^,^^69^ Aligning the route of administration with subgroup-specific patterns of failure may position CSF-directed delivery as a rational, compartment-matched strategy in metastatic MB.

#### Nanoparticle-based delivery systems

In MB, nanoscale delivery platforms are increasingly explored to address the pharmacokinetic and biological limitations associated with free active pharmaceutical ingredients. Rapid CSF turnover, which occurs approximately four to five times daily in humans, can reduce drug residence time within the leptomeningeal compartment. In addition, many lipophilic chemotherapeutics are substrates of P-gp and other ABC transporters, which actively restrict parenchymal drug accumulation.^75^

Nanotechnology-based systems, including lipid and polymeric nanocarriers as well as ligand-directed nanobiologic vectors, provide versatile strategies to modify drug-microenvironment interactions. By encapsulating therapeutic payloads within nanoscale structures, these platforms can alter biodistribution profiles, facilitate transport via endocytic or receptor-mediated pathways rather than passive diffusion alone, and enhance penetration within tumor-associated vasculature. Their nanoscale dimensions enable improved dispersion through interstitial spaces, while surface engineering approaches such as PEGylation or ligand functionalization can optimize tissue distribution and cellular uptake. In addition, nanocarriers can enhance payload stability, enable controlled-release kinetics, and reduce recognition by efflux transporters, thereby improving intratumoral retention and spatial distribution compared with unencapsulated agents.^75^

Within this framework, liposomal formulations and ligand-directed phage platforms are particularly relevant to MB. Liposomes represent one of the most clinically advanced nanocarrier technologies and have demonstrated improved intratumoral distribution and pharmacokinetic stability in preclinical brain tumor models.^76^ On the other hand, phage-guided systems leverage receptor-specific vascular targeting and intracellular gene expression to achieve tumor-selective delivery.^3^

#### Liposomal delivery systems

Liposomal formulations represent one of the most clinically advanced classes of nanoparticle systems and have been extensively evaluated in oncology. Their biocompatibility, high drug-loading capacity, and ability to shield encapsulated agents from rapid clearance make them attractive vehicles for chemotherapeutics and targeted agents.^77^^,^^78^

In MB, MacDonald^79^ explored a novel therapeutic approach for treating SHH MB, which is a particularly aggressive form of MB. The researchers focused on using imipramine blue (IB), a molecule with antitumor properties, encapsulated in a liposome to create a delivery system called Liposome IB (Lipo IB). This liposomal nanoparticle was engineered to cross the BBB and preferentially target tumor cells within the brain, which is a critical challenge in treating brain tumors.^79^^,^^80^

The results of this study demonstrated that Lipo IB effectively decreased cell viability and migration in SHH MB both *in vitro* and *in vivo*. The treatment resulted in significant tumor growth inhibition, reduced tumor volume (including complete tumor regression), and improved survival in mice with minimal toxicity. Lipo IB was particularly effective as a monotherapy, suggesting that it could potentially be developed for clinical applications, offering a promising alternative to existing therapies for high-risk SHH MB, which often resist conventional treatments. The liposomal delivery system provided a key advantage in overcoming the BBB, ensuring that the therapeutic molecule was efficiently delivered to the tumor site. This liposomal formulation enhances the lipophilicity of IB, improving its CNS penetration and uptake by tumor cells.^79^

#### Transmorphic phage-guided systemic gene delivery

Gene therapy for cancer treatment has long been explored, with viral vectors commonly used to deliver therapeutic genes.^80^ In particular, bacteriophages modified to target mammalian cells have emerged as a promising option for cancer gene therapy due to their ability to avoid native tropism and selectively deliver therapeutic agents to tumors.^3^ Bacteriophages, especially filamentous M13 bacteriophages, have been engineered for gene delivery, often combined with other viruses such as adeno-associated virus (AAV) to improve transgene expression. A hybrid model, known as the AAV phage vector, has shown efficient tumor targeting through receptor-mediated ligand display on their capsids.^80^^,^^81^^,^^82^

Recent innovations have led to the development of the TPA system, which offers an enhanced gene delivery efficiency and the ability to accommodate larger DNA inserts. These vectors, particularly when designed to display tumor-specific ligands such as RGD4C, have demonstrated selective homing to tumors in preclinical models of MB.^82^^,^^83^ By displaying the RGD4C ligand, this system facilitates binding to tumor vasculature, enabling localized gene delivery, while minimizing systemic exposure.

This targeted approach has shown promise in addressing challenges associated with BBB-restricted drug delivery through receptor-mediated vascular targeting.^3^

Using the RGD4C.TPA.TNFα system, which targets tumor cells while sparing healthy tissues, has proven effective in delivering the tumor necrosis factor alpha (TNFα) to MB cells, promoting apoptosis and enhancing the efficacy of chemotherapeutic agents like cisplatin.^3^ The therapeutic potential of TNFα as a gene therapy for brain tumors was tested for the first time in this context. TNFα has been found to downregulate vascular endothelial cadherin, thereby increasing the permeability of the tumor vasculature. This enhanced permeability allows for improved delivery and uptake of chemotherapeutic agents like melphalan and doxorubicin, which are otherwise hindered by the BBB. Studies suggest that TNFα′s ability to increase vascular leakiness can make the tumor more sensitive to chemotherapy, thereby synergizing with standard treatments to boost efficacy and reduce toxicity.^3^

Overall, the RGD4C.TPA.TNFα system represents an investigational targeted strategy for MB with the potential for more effective, safer, and less-invasive treatment options that may complement existing therapeutic approaches. While preclinical models demonstrate therapeutic efficacy, further evaluation in clinical trials will be required to establish safety and clinical benefit in patients with MB.

#### Focused ultrasound therapy

FUS is an emerging non-invasive therapeutic technology with significant potential for treating brain tumors. It works by precisely directing beams of ultrasonic energy to deep brain tissues, including tumors, while sparing surrounding healthy tissue. One of its key advantages is its ability to temporarily open the BBB.^83^^,^^84^ In a study, FUS facilitated the targeted delivery of fluorescently labeled immunoglobulin G to both the cerebellum and MB tumors, significantly enhancing antibody delivery by 1.3 times to the cerebellum and 1.5 times to the tumors compared with controls. Importantly, the procedure did not cause any tissue damage, and safety was confirmed through imaging, histology, and monitoring of motor and cardiorespiratory functions, all of which showed no adverse effects.^4^

Highly vascularized tumors, such as WNT subgroup MB, may particularly benefit from FUS technology. This approach involves the systemic administration of microbubbles and therapeutic agents to enhance drug delivery.^4^ While FUS can transiently disrupt the BBB, it has been reported to suppress efflux transporters, which may improve drug accumulation and retention within the brain parenchyma. Conversely, convection-enhanced delivery (CED) is particularly effective for tumors characterized by low vascular density and an intact BBB, such as diffuse intrinsic pontine glioma. These conditions reduce the risk of infused drugs diffusing away from the targeted tumor site.^4^^,^^84^

**Figure 4:**
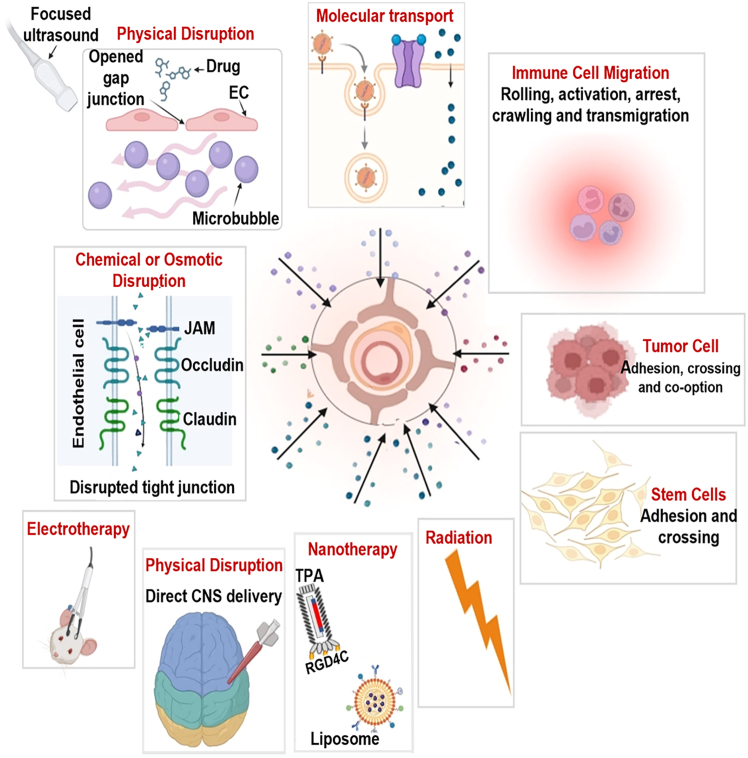
Key mechanisms for crossing the BBB and BBTB Cell-mediated BBB/BBTB disruption, molecular and nanotherapeutic strategies, and physical methods for direct delivery. EC, endothelial cell; TPA, transmorphic phage/AAV.

In addition, when combined with treatments like radiation or chemotherapy, FUS has been shown in preclinical studies to enhance the effectiveness of these therapies, while minimizing systemic side effects. FUS improves drug delivery by enhancing the penetration of therapeutic agents, including chemotherapy and immunotherapy, when administered intravenously or through carriers like TPA, liposomes, or microbubbles.^85^^,^^86^^,^^87^^,^^88^ This method enables precise tumor targeting and higher local drug concentrations, reducing the impact on the surrounding healthy tissue.^87^ Although FUS is still undergoing investigation and is not yet widely approved, ongoing studies continue to explore its potential as a future treatment option for high-risk brain tumors, demonstrating promise in improving both treatment precision and patient outcomes (Figure 4).

#### BBB opening by low PEFs

The study by Cooper,^89^ explored the use of low PEFs, referred to as barrier-disrupting fields (BDF), as an innovative approach to enhance drug delivery to the brain, particularly for brain tumors. BDF employs low-intensity PEFs to temporarily disrupt the BBB, enabling therapeutic agents to bypass this critical obstacle. This method effectively increased the brain penetration of doxorubicin, a chemotherapeutic drug, overcoming resistance associated with efflux pumps like P glycoprotein.^89^^,^^90^

The researchers confirmed the efficacy of BDF using delayed contrast MRI, which visualized the extent of BBB disruption. However, the drug distribution within the brain was uneven, with higher concentrations in certain regions due to variability in electric field strength. Despite this limitation, the approach achieved therapeutic concentrations, offering potential improvements in treatment outcomes.^89^

In addition, Tanori^92^ explored the use of microsecond PEFs (μsPEFs) as a novel therapeutic approach to target cancer stem cells (CSCs) in MB. Human MB D283Med cells were treated with μsPEF *in vitro* and then injected subcutaneously into mice. The researchers found their PEF protocol (0.3 MV/m, 40 μs, 5 pulses) selectively induced irreversible membrane permeabilization and apoptosis in MB CSCs. Furthermore, combining μsPEF with radiation therapy showed a synergistic effect in inhibiting tumor growth.^91^ These findings suggest that μsPEF may be a promising therapeutic strategy for MB, offering a potential to improve patient outcomes. The results highlight the potential of PEF as a non-invasive and precise strategy to enhance the effectiveness of radiotherapy, while sparing healthy brain tissue and effectively crossing the BBB, paving the way for improved treatment of CSC-driven brain tumors.

#### Convection-enhanced delivery

CED represents a mechanical bypass of the BBB by utilizing surgically implanted microcatheters to infuse therapeutics directly into the brain parenchyma. Unlike diffusive therapies that are governed by concentration gradients and Fick’s law, CED establishes a positive pressure gradient to drive bulk flow through the interstitial space. This mechanism, described by Darcy’s law, allows for a more homogeneous drug distribution across several centimeters and is largely independent of the molecular weight of the infusate, making it a promising platform for delivering large-volume macromolecules such as liposomes or targeted toxins.^75^

Despite these technical advantages, several factors limit the broad application of CED in the MB paradigm. First, while CED is highly effective for localized, solid tumor masses, it is physically constrained by the volume that can be treated with a single catheter trajectory. This represents a significant mismatch for MB subgroups, particularly group 3 and group 4, which frequently present with widespread leptomeningeal dissemination across the neuraxis. Because CED is a focal delivery method, achieving total coverage of the spinal axis and brain surface is technically impractical compared with surface-directed intrathecal therapies. Furthermore, the high cellular density of the cerebellum can lead to elevated ISF pressure, which increases the risk of backflow or reflux along the catheter-brain interface, potentially resulting in sub-therapeutic drug concentrations at the target site and off-target neurotoxicity in unintended regions.^75^ The invasive nature of repeated stereotactic procedures also poses a significant burden in pediatric care, leading many clinicians to prioritize less-invasive alternatives like FUS for barrier modulation. Nevertheless, CED remains a critical tool for recurrent, localized disease, as evidenced by ongoing clinical trials evaluating the panobinostat-cyclodextrin complex MTX110 (NCT04315064), where precise parenchymal saturation is required to overcome restrictive barrier features.

### Discussion and future directions

The central challenge in MB therapy is not only identifying effective therapeutic agents but also ensuring that those agents achieve anatomically appropriate and durable exposure within the relevant CNS compartment. The BBB, BBTB, and BCSFB are physiologically distinct structures that impose compartment-specific constraints on drug distribution. While the BBB and BBTB regulate parenchymal penetration, the BCSFB governs exchange between systemic circulation and the CSF, creating pharmacokinetic differences that are often overlooked in drug development.

Importantly, patterns of recurrence in MB are strongly subgroup dependent and provide insight into where therapeutic failure occurs anatomically. SHH MB most commonly recurs locally within the posterior fossa, consistent with insufficient intraparenchymal drug exposure or heterogeneous intratumoral distribution. In contrast, group 3 and group 4 tumors frequently relapse with metastatic or leptomeningeal dissemination, implicating inadequate therapeutic coverage of the CSF and neuraxis rather than failure of local tumor control alone.^10^^,^^12^ These recurrence patterns argue against a uniform CNS delivery strategy and instead support a compartment-matched framework.

Within this context, delivery strategies that enhance parenchymal penetration, such as barrier modulation or biologically targeted vascular approaches, may be particularly relevant for SHH tumors that retain restrictive BBB characteristics. Conversely, CSF-directed strategies, including intraventricular administration, are biologically aligned with group 3 and group 4 disease, where leptomeningeal relapse predominates. However, rapid CSF turnover and limited parenchymal penetration remain major pharmacokinetic constraints, highlighting the need for sustained release and spatially optimized delivery systems.^25^

Barrier integrity in MB is also dynamic rather than static. Radiotherapy and chemotherapy have been shown to alter vascular permeability and perivascular structures associated with CSF interstitial exchange, suggesting that treatment itself may modify drug distribution properties over time.^74^ These findings highlight the importance of integrating temporal barrier changes into both preclinical modeling and clinical trial design.

A key implication for translational research is the need to align preclinical pharmacokinetic endpoints with clinically dominant sites of failure. Whole brain homogenate measurements or plasma concentrations may substantially overestimate effective exposure within tumor-adjacent parenchyma or CSF compartments. Subgroup-specific assessment, measuring intratumoral distribution in SHH models and CSF/spinal exposure in group 3 and group 4 models, may improve predictive accuracy and reduce late-stage translational failure.

Ultimately, effective drug delivery in MB requires moving beyond a one-size-fits-all paradigm toward precision, compartment-matched strategies informed by molecular subgroup, barrier biology, and recurrence anatomy. By embedding delivery considerations early in therapeutic development and explicitly distinguishing parenchymal and CSF targets, future approaches may improve both efficacy and long-term safety in children with MB.

## Acknowledgments

The authors wish to thank the 10.13039/100013790Brain Research UK for their support with the 202021-34 award and 10.13039/501100001273Children with Cancer UK for the 16-230 and 2013/147 grants.

## Author contributions

The first draft of the manuscript was written by K.B., S.L., and A.H. All authors participated in the subsequent revision and critical review of the content. Intellectual and conceptual contributions were provided by K.S. and R.E. All authors have read and approved the final version of the manuscript.

## Declaration of interests

K.S. and A.H. are named inventors on a granted patent covering the TPA vector.

## Supplemental information

Table S1. Recent pharmacological therapies in preclinical *in vivo* efficacy studies for medulloblastoma

Table S2. Prominent approaches in treating medulloblastoma: A review of recent clinical trials (Ref: clinicaltrials.gov)
